# Knowledge, Attitudes, and Practices Regarding Marburg Virus Disease Among Healthcare Workers in Dilla University Teaching Hospital, Ethiopia: A Cross‐Sectional Study

**DOI:** 10.1155/cjid/7912748

**Published:** 2026-10-25

**Authors:** Abel Desalegn Demeke, Takla Tamir, Mesfin Abebe, Migbaru Endawoke, Atirsaw Shimekaw Goshu, Samuel Jigso Dube, Endris Seid Amede, Wondimagegn Wolde Eba, Aboma Tsegaye Molatu, Alemayehu Yeshiwas, Mequanint Ayehu Akele, Alem Bayable

**Affiliations:** ^1^ Department of Nursing College of Medicine and Health Sciences Dilla University Dilla Ethiopia du.edu.et; ^2^ Department of Midwifery College of Medicine and Health Sciences Dilla University Dilla Ethiopia du.edu.et; ^3^ Department of Psychiatry College of Medicine and Health Sciences Dilla University Dilla Ethiopia du.edu.et; ^4^ Department of Medical Laboratory Science College of Health Science Oda Bultum University Chiro Ethiopia obu.edu.et; ^5^ School of Medicine College of Medicine and Health Sciences Dilla University Dilla Ethiopia du.edu.et

**Keywords:** attitude, Ethiopia, knowledge, Marburg virus disease, practice

## Abstract

**Background:**

Healthcare workers are a first contact for patients with infectious diseases and may be at occupational risk during hemorrhagic fever outbreaks. The Marburg virus disease (MVD) outbreak in southern Ethiopia, ongoing during data collection in December 2025, highlighted the need to assess healthcare workers’ knowledge, attitudes, and practices regarding MVD. Therefore, this study assessed knowledge, attitudes, and practices and examined factors associated with practice among healthcare work.

**Methods:**

A cross‐sectional study of 238 healthcare workers at Dilla University Teaching Hospital was conducted from December 1 to December 22, 2025. Descriptive statistics, frequencies, and percentages summarized participant characteristics and KAP responses. Binary logistic regression examined factors associated with good practice. Variables with *p* value ≤ 0.25 in bivariable analysis were included in the multivariable model. Adjusted odds ratios (AORs) with 95% confidence intervals (CIs) were calculated, and significance was set at *p* < 0.05.

**Results:**

About 51.7%, 57.1%, and 55.0% of participants had good knowledge, positive attitudes, and good practice toward MVD, respectively, based on study‐specific cutoffs. Most identified direct contact with blood and body fluids as an MVD transmission route (92.0%). However, gaps were observed regarding environmental risk factors and MVD distribution and origin. Most recognized reporting suspected cases (91.2%), avoiding contact with infected individuals or their possessions (91.2%), and using personal protective equipment (87.8%). In multivariable analysis, modification of working habits from concern about acquiring MVD (AOR = 2.19, 95% CI: 1.21–3.99) and good MVD knowledge (AOR = 1.96, 95% CI: 1.13–3.39) were positively associated with good practice.

**Conclusion:**

Findings indicate gaps in MVD‐related knowledge and infection prevention among healthcare workers at Dilla University Teaching Hospital. Targeted competency‐based training, simulation, IPC resources, and facility‐level preparedness systems are warranted to strengthen readiness for MVD and other viral hemorrhagic fever events.

**What This Study Adds**
• Provides evidence on knowledge, attitudes, and practices regarding Marburg virus disease among healthcare workers in southern Ethiopian teaching‐hospital settings.
• Identifies knowledge gaps in environmental risk factors, zoonotic transmission, and geographic distribution of Marburg virus disease.
• Examines practice‐associated factors, including knowledge and modified work habits due to concern about acquiring MVD.
• Provides facility‐level evidence informing targeted training, infection prevention and control preparedness, and outbreak‐response planning in similar settings.

## 1. Introduction

Viral hemorrhagic fevers (VHFs) are illnesses caused by viruses from various families. Marburg virus disease (MVD) is a severe, potentially lethal VHF caused by Marburg virus (MARV), an *orthomarburgvirus* in the Filoviridae family. Although Ebola virus disease and MVD are caused by different viruses in the same family, they can exhibit similar symptoms. Marburg virus is linked to fruit bats, its natural reservoir [1–3]. Human infection occurs through contact with infected animals and subsequent human‐to‐human transmission via direct contact with infected individuals’ blood, secretions, organs, or bodily fluids, as well as contaminated materials via broken skin or mucous membranes. Transmission may also occur during unsafe burials. Consequently, healthcare workers may face occupational risk when treating suspected or confirmed cases without infection prevention and control (IPC) measures [1, 2, 4].

Outbreaks of MVD have occurred in African countries and, historically, outside Africa. The first outbreaks occurred in 1967 in Germany and Yugoslavia, involving laboratory workers exposed to African green monkeys imported from Uganda. Subsequent outbreaks and imported cases were reported in South Africa, Kenya, the Democratic Republic of the Congo, Angola, Uganda, the Netherlands, the United States, Guinea, Ghana, Equatorial Guinea, Tanzania, and other nations [2, 4–6]. On September 27, 2024, Rwanda confirmed its first MVD outbreak, the first documented occurrence in Rwanda, not the first global outbreak. The outbreak was declared concluded on December 20, 2024, following documentation of 66 confirmed cases and 15 fatalities [7, 8]. Tanzania encountered a second MVD outbreak in the Kagera Region in January 2025, after its initial 2023 outbreak there. The 2025 outbreak comprised two confirmed and eight probable cases, all fatal, and was declared concluded on March 13, 2025 [9].

Symptoms of MVD include the initial manifestation of sudden high fever, severe headache, and weakness, followed by vomiting, diarrhea, rash, and bleeding in severe cases, which resemble illnesses caused by other viruses [1, 6]. The average case fatality rate of MVD is 50%, ranging from 24% to 88%. Currently, no curative treatment or vaccine has been approved; therefore, supportive care management such as rehydration and symptomatic treatment, accompanied by early detection, IPC, and safe burial practices, is the preferred approach [1, 10, 11].

HCWs are the first point of contact for patients with infectious disease, and the risk of contracting infection was increased due to various factors, including a failure to recognize prospective cases, poor infection control practice adherence, and insufficient healthcare infrastructure [12, 13]. A recent study reported that poor infection control practices were found among HCWs as compared to non‐HCWs [14]. Another study conducted among healthcare professionals in Sierra Leone depicted a lack of knowledge about symptom differences in Ebola and MVD [15].

In Ethiopia, the initial case of MVD manifested symptoms on October 23, 2025, with laboratory confirmation on November 14, 2025. The outbreak was declared concluded on January 26, 2026, following a 42‐day period without new confirmed cases. By the outbreak’s conclusion, 19 cases had been documented, including 14 laboratory‐confirmed cases, of which nine resulted in fatalities, and five probable cases, all of whom succumbed to the disease [16]. The recent outbreak of MVD in southern Ethiopia underscores the need to evaluate healthcare workers’ readiness to identify, prevent, and manage MVD cases. While studies in Ethiopia have explored healthcare workers’ knowledge, attitudes, and preparedness for MVD outbreak response [17, 18], evidence remains scarce regarding factors influencing self‐reported practices across settings. Notably, facility‐level data from teaching hospitals in southern Ethiopia are lacking. Consequently, this study aimed to evaluate healthcare workers’ knowledge, attitudes, and practices concerning MVD and investigate factors associated with practices at Dilla University Teaching Hospital in southern Ethiopia.

## 2. Materials and Methods

### 2.1. Study Design, Period, and Settings

The study population comprised healthcare workers employed at Dilla University Teaching Hospital. Eligible participants were those involved in direct patient care, including doctors, nurses, midwives, pharmacists, laboratory professionals, psychiatrists and/or other designated mental‐health professionals, anesthetists, public health officers, and optometrists. Participants were eligible if working at the hospital during the study period and providing informed consent. Healthcare workers without direct patient‐care responsibilities, those on leave during the data‐collection period, temporary staff, interns, trainees, and other categories excluded from the staff list used for recruitment were.

### 2.2. Study Population and Eligibility Criteria

The study population comprised healthcare workers at the Dilla University Teaching Hospital during the study period. Eligible participants who provided direct patient care included medical doctors, nurses, midwives, pharmacists, laboratory professionals, psychiatrists and/or other designated mental health professionals, anesthetists, public health officers, and optometrists. The eligibility criteria were working at the hospital during the study period and providing informed consent. Healthcare workers without direct patient‐care responsibilities, individuals on leave during the data‐collection period, temporary staff, interns, trainees, and other categories not included in the hospital staff list used for recruitment were.

### 2.3. Sample Size Determination and Sampling Technique

In the study country, as far as our searching goes, there was no study done previously; the sample size was calculated using a 50% assumed prevalence of good knowledge, attitude, or practice related to MVD by a single population proportion formula. Finally, an additional 10% was added to account for potential nonrespondents.

(1) nu=Zα22p1−pd2.

*n* *u* = (1.96)^2^0.5(1–0.5)/0.05^2^ = 384. However, the actual number of healthcare professionals at Dilla University Teaching Hospital, which was the source population for the study, was 513 and less than 10,000. Therefore, the following correction formula for a finite population was employed.

*n* *c* = *n* *u*/(1 + (*n* *u*)/*N*) = 384/(1 + 384/513) = 220. After adding 10%, the minimum sample size became 242.

Where *n* *u* = uncorrected sample size; *n* *c* = corrected sample size; *α* = level of significance (set at 0.05); *Z* *α*/2 = *a* standardized normal test with *α* level of significance (it is equivalent to 1.96 with the corresponding 95% CI for the given value); *d* = margin of error; *p* = estimated population proportion; *N* = total number of source population.

To recruit a participant, a simple random sampling technique was employed by using the list of health professionals as a frame.

### 2.4. Data Collection

The validated English data collection tool was adopted from a previously conducted study [14]. A voluntary‐response sampling approach was used. The online questionnaire link was disseminated to eligible healthcare workers via institutional Telegram channels. Eligible healthcare workers receiving the survey invitation and providing informed consent could participate. Participants completing the questionnaire constituted the final study sample.

### 2.5. Operational Definition

*Health care workers:* Healthcare workers were professionals in direct patient care at Dilla University Teaching Hospital, including doctors, nurses, midwives, pharmacists, laboratory professionals, psychiatrists and/or other mental health professionals, anesthetists, public health officers, and optometrists.

*Good knowledge:* Participants were classified as possessing good knowledge if their scores on the knowledge items exceeded the study‐specific mean [14].

*Poor knowledge:* Participants are considered to have limited knowledge if their responses to the knowledge item questions are at or below the mean score [14].

*Positive attitude:* Participants exhibit a positive attitude if their responses to the attitude item questions exceed the mean [14].

*Poor attitude:* Participants demonstrate a poor attitude if their responses to the attitude item questions are equal to or below the mean [14].

*Good practice:* Participants engage in good practice if their responses to the practice item questions surpass the mean [14].

*Poor practice:* Participants exhibit poor practice if their responses to the practice item questions are equal to or below the mean [14].

The study classifications were derived from mean scores, not externally validated competency thresholds. Consequently, these categories reflect classifications within the sample and should not be construed as evidence of achieving or failing an objective competency standard.

### 2.6. Statistical Analysis

Data were entered, checked, cleaned, and analyzed using IBM SPSS Statistics Version 26.0. Descriptive statistics, including frequencies and percentages, summarized participants’ sociodemographic characteristics and responses to knowledge, attitude, and practice items. Binary logistic regression analysis examined factors associated with good practice regarding MVD. Good practice was coded as the outcome variable, with good practice coded as 1 and poor practice as 0. In bivariable analysis, each explanatory variable was assessed against the practice outcome. Variables with *p* value ≤ 0.25 were candidates for the multivariable logistic regression model.

The multivariable model estimated adjusted odds ratios (AORs) with 95% CIs. Categorical variables were entered with reference categories specified in the regression model and tables. Statistical significance was considered at *p* < 0.05. Multicollinearity among explanatory variables was assessed before fitting the final model using variance inflation factor (VIF) and tolerance. Model goodness‐of‐fit was assessed using the Hosmer–Lemeshow test, with the statistic and *p* value reported. Because this was a cross‐sectional study, associations identified in regression analysis were interpreted as adjusted associations, not evidence of temporal or causal relationships.

## 3. Result

### 3.1. Sociodemographic Characteristics

A total of 238 healthcare workers participated in the study, yielding a 98.3% response rate. Most participants were male (74.8%). Half of participants, 126 (52.9%), were aged 30–39 years. Regarding work experience, 138 (58.0%) had 6–10 years of professional experience (Table 1).

**TABLE 1:** Sociodemographic characteristics of participants.

Characteristics	Category	Frequency
Age (Y)	20–29	105 (44.1)
	30–39	126 (52.9)
	≥ 40	7 (2.9)

Sex	Male	178 (74.8)
	Female	60 (25.2)

Marital status	Single/divorced/widowed	129 (54.2)
	Married	109 (45.8)

Profession	Nurse (all specialty)	126 (52.9)
	Medical doctor (all specialty)	32 (13.4)
	Psychiatry	21 (8.8)
	Midwifery	21 (8.8)
	Laboratory	23 (9.7)
	Others^∗^	15 (6.3)

Experience (Y)	1–5	78 (32.8)
	6–10	138 (58)
	> 10	22 (9.2)

*Note:* Y; years, others ^∗^; pharmacists, anesthetists, public health officers, and optometrist.

### 3.2. Awareness Toward MVD

In this study, more than half of the participants, 157 (66%), heard about MVD before. When it comes to mode of transmission, 132 (55.5%) participants answered correctly that MVD is transmitted through direct contact with Bat. Nearly half of the participants, 116 (48.7), had physical body contact with others more than five times per day (Table 2).

**TABLE 2:** Awareness of participant toward MVD.

Awareness about MVD	Category	Frequency
Heard about MVD before	No	81 (34)
	Yes	157 (66)

MVD is easily transmitted through direct contact with	Bats	132 (55.5)
	Cattle	15 (6.3)
	Mosquitoes	35 (14.7)
	Sheep and goats/pigs/poultry	18 (7.6)
	Rodents	13 (5.5)
	I do not know	25 (10.5)

Performed modifications of working habits because of fears about getting MVD	No	74 (31.1)
	Yes	164 (68.9)

Frequency of physical bodily contact with others (e.g., handshake)	Never	37 (15.5)
	1–3 times daily	45 (18.9)
	4–5 times per day	40 (16.8)
	> 5 times per day	116 (48.7)

Abbreviation: MVD, Marburg virus disease.

### 3.3. Knowledge Toward MVD

The participant who scored above the mean of the knowledge questions were 123 (51.7%), which was considered as having good knowledge. Out of the total domain of knowledge, “Marburg is easily transmitted from human to human by direct contact with blood and body fluids” was the most correctly answered domain, as 219 (92%) of participants responded “Yes.” Significantly more respondents, 88.7%, think that individuals at risk for contacting Marburg are hunters, health workers, and those in contact with patient travelers to epidemic areas. Nonetheless, 7.1% expressed as not, and 4.2% do not know this idea (Table 3).

**TABLE 3:** Knowledge of participant toward MVD.

Knowledge question	Category	Frequency
MVD is known as Marburg hemorrhagic fever	Yes	214 (89.9)
	No	3 (1.3)
	I do not know	21 (8.8)

MVD outbreaks have originated in Africa	Yes	127 (53.4)
	No	64 (26.9)
	I do not know	47 (19.7)

Marburg is easily transmitted from human to human by direct contact with blood and body fluids	Yes	219 (92)
	No	3 (1.3)
	I do not know	16 (6.7)

MVD can spread through food	Yes	78 (32.8)
	No	109 (45.8)
	I do not know	51 (21.4)

Individuals at risk for contacting Marburg are hunters, health workers, and those contact with patient travelers to epidemic areas	Yes	211 (88.7)
	No	17 (7.1)
	I do not know	10 (4.2)

MVD causes severe bleeding	Yes	217 (91.2)
	No	3 (1.3)
	I do not know	18 (7.6)

Severe diarrhea and vomiting are among the MVD symptoms	Yes	167 (70.2)
	No	27 (11.3)
	I do not know	44 (18.5)

MVD ends by death in half of the patients	Yes	193 (81.1)
	No	11 (4.6)
	I do not know	34 (14.3)

Avoiding mines and caves where fruit bats thrive can prevent the spread of the disease	Yes	164 (68.9)
	No	23 (9.7)
	I do not know	51 (21.4)

Avoiding contact with an infected person or his objects can prevent the spread of the disease	Yes	217 (91.2)
	No	0 (0)
	I do not know	21 (8.8)

Reporting symptoms of MVD to local authorities is important to prevent further disease transmission	Yes	217 (91.2)
	No	18 (7.8)
	I do not know	3 (1.3)

Wearing personal protective equipment like gloves could prevent the disease spread	Yes	209 (87.8)
	No	7 (2.9)
	I do not know	22 (9.2)

Avoiding the funeral of MVD‐infected patients prevents transmission of the disease from the dead person	Yes	182 (76.5)
	No	31 (13)
	I do not know	25 (10.5)

Knowledge	Good	123 (51.7)
	Poor	115 (48.3)

### 3.4. Attitude Toward MVD

Of the 238 participants, 136 (57.1%) had a positive attitude toward MVD based on the study‐specific mean cutoff. In Table 4, 77 (32.4%) participants strongly agreed they would receive a Marburg virus vaccine if available, while 65 (27.3%) strongly agreed they trusted information about MVD from scientific experts (Table 4).

**TABLE 4:** Attitude of participants toward MVD.

Attitude question	Category	Frequency
I should learn more about MVD	Totally disagree	15 (6.3)
	Disagree	1 (0.4)
	Neutral	13 (5.5)
	Agree	118 (49.6)
	Totally agree	91 (38.2)

People should be tested when they are in contact with someone infected	Totally disagree	16 (6.7)
	Disagree	1 (0.4)
	Neutral	4 (1.7)
	Agree	92 (38.7)
	Totally agree	125 (52.5)

I think MVD prevention and control measures are important	Totally disagree	25 (10.5)
	Disagree	2 (0.8)
	Neutral	4 (1.7)
	Agree	74 (31.1)
	Totally agree	133 (55.9)

I should take Marburg virus vaccine if it is available	Totally disagree	24 (10.1)
	Disagree	21 (8.8)
	Neutral	25 (10.5)
	Agree	91 (38.1)
	Totally agree	77 (32.4)

I worry MVD will become a new pandemic	Totally disagree	19 (8)
	Disagree	19 (8)
	Neutral	49 (20.6)
	Agree	97 (40.8)
	Totally agree	54 (22.7)

I do trust the information about MVD from scientific experts	Totally disagree	25 (10.5)
	Disagree	8 (3.4)
	Neutral	45 (18.9)
	Agree	95 (39.9)
	Totally agree	65 (27.3)

I should take more hygienic measures like frequent handwashing	Totally disagree	0 (0)
	Disagree	23 (9.7)
	Neutral	90 (37.8)
	Agree	109 (45.8)
	Totally agree	16 (6.7)

Attitude	Positive	136 (57.1)
	Negative	102 (42.9)

### 3.5. Practice Toward MVD

Of the 238 participants, 131 (55.0%) had good practice and 107 (45.0%) poor practice based on the study‐specific mean cutoff. Regarding hand hygiene, 50 (21.0%) participants reported always washing hands for at least 20 s using soap and water or an alcohol‐based hand sanitizer, 49 (20.6%) often, 89 (37.4%) sometimes, 39 (16.4%) rarely, and 11 (4.6%) never. In addition, 57 (23.9%) participants always wore a face mask (Table 5).

**TABLE 5:** Practice of participants toward MVD.

Practice question	Category	Frequency
How often do you wash your hands at least 20 s with soap and water and alcohol‐based hand sanitizers?	Never	11 (4.6)
	Rarely	39 (16.4)
	Sometimes	89 (37.4)
	Often	49 (20.6)
	Always	50 (21)

How often do you wash and disinfect surfaces and utensils?	Never	7 (2.9)
	Rarely	49 (20.6)
	Sometimes	79 (33.2)
	Often	44 (18.5)
	Always	59 (24.8)

How often do you wear a facemask nowadays?	Never	27 (11.3)
	Rarely	37 (15.5)
	Sometimes	90 (37.8)
	Often	27 (11.3)
	Always	57 (23.9)

How often do you wear gloves nowadays?	Never	16 (6.7)
	Rarely	27 (11.3)
	Sometimes	57 (23.9)
	Often	49 (20.6)
	Always	89 (37.4)

Practice	Good	131 (55)
	Poor	107 (45)

### 3.6. Factors Associated With Healthcare Workers’ Practice Toward MVD

In the multivariable logistic regression model, modification of working habits due to concern about acquiring MVD and knowledge were associated with good practice. Participants reporting modification of working habits due to concern about acquiring MVD had higher odds of good practice than those without modification (AOR = 2.19, 95% CI: 1.21–3.99). Similarly, participants with good MVD knowledge had higher odds of practice than those with poor knowledge (AOR = 1.96, 95% CI: 1.13–3.39). These findings are cross‐sectional associations and should not be interpreted as evidence that fear or knowledge caused better practice (Table 6).

**TABLE 6:** Determinants of healthcare workers’ practice toward Marburg virus disease: multivariable logistic regression analysis.

Variables	Category	Practice		COR (95% CI)	AOR (95% CI)	*p* value
		Good (%)	Poor (%)			
Performed modifications of working habits because of fears about getting MVD	No	29 (39.2)	45 (60.8)	1	1	0.010
	Yes	102 (62.2)	62 (37.8)	2.55 (1.45, 4.49)	2.19 (1.21, 3.99)^∗^	

Knowledge toward MVD	Poor	52 (45.2)	63 (54.8)	1	1	0.017
	Good	79 (64.2)	44 (35.8)	2.18 (1.29, 3.66)	1.96 (1.13, 3.39)^∗^	

^∗^The corresponding 95% confidence intervals for significant predictors.

## 4. Discussion

In their work, healthcare workers may have contact with patients and potentially infectious fluids and require preparedness to recognize and prevent MVD transmission. This study assessed MVD knowledge, attitudes, and practices and examined factors associated with practice among healthcare workers at Dilla University Teaching Hospital in southern Ethiopia. The findings provide evidence from a southern Ethiopian teaching‐hospital setting and complement studies in other Ethiopian settings.

51.7% of healthcare workers were classified as having good knowledge of MVD based on the study‐specific mean cutoff. This indicates heterogeneous knowledge in the population, with 48.3% at or below the sample mean. However, because classification was based on the study‐specific mean rather than an externally validated competency threshold, these findings should not be interpreted to indicate that participants lacked a minimum knowledge level. Specific gaps were observed in environmental risk factors and MVD geographic distribution. For example, 68.9% of participants recognized caves and mines as fruit bat environments, while 53.4% correctly identified Africa as the source of most MVD outbreaks. These findings are consistent with studies reporting healthcare worker knowledge gaps in the epidemiology and transmission of VHFs [17, 19, 20, 21, 22]. This knowledge gap matters because recognizing environmental and zoonotic exposure settings is key for risk assessment, early recognition, and prevention of MVD transmission.

On the other hand, the high degree of awareness regarding prevention and transmission is one of the study’s strongest points. 91.2% of participants recognized the significance of avoiding contact with an infected person’s possessions, and a surprising 92% of participants correctly identified that MVD is spread through direct contact with bodily fluids. This is consistent with a study conducted in Nigeria [23], where the majority of health professionals correctly identified bodily fluids as the main spread route. This is probably a result of the repeated focus on infection control and prevention during previous outbreaks in Africa, implying core infection prevention control (IPC) messaging has been absorbed. The fact that over 90% of our participants recognized MVD causes severe bleeding and that 81.1% understood its high mortality rate is a strength. This fear factor often encourages greater adherence to preventative measures. The majority of participants acknowledged the importance of reporting suspected cases (91.2%), avoiding contact with infected people (91.2%), and wearing personal protective equipment (87.8%) as important preventive measures. High recognition of key infection prevention measures is consistent with reports on IPC measures among healthcare professionals during the 2024 MVD outbreak response in Rwanda [24]. Strong reporting and PPE use awareness points to a potentially well‐established infection prevention culture at the hospital.

Regarding attitude, 57.1% of participants had a positive attitude toward MVD based on the study‐specific mean cutoff. This indicates that over half scored above the sample mean on the attitude items. The result is comparable with findings from studies among healthcare workers in India, Uganda, and Ethiopia [17, 19, 25]. Differences between studies may reflect variation in populations, timing relative to outbreaks, measurement instruments, and methods used to define positive attitudes.

In this study, 55.0% of healthcare workers were classified as having good practice using the study‐specific mean cutoff. Because the cutoff was derived from sample distribution rather than an externally validated competency standard, the finding should be interpreted as a relative classification in the study population rather than evidence of objectively adequate or inadequate preparedness. This finding is consistent with evidence from Ethiopia, where a systematic review reported an overall hand hygiene compliance rate of only 38%. The review further demonstrated that compliance was strongly associated with institutional factors, including the availability of hand hygiene supplies, implementation of hand hygiene promotion programs, and regular staff training [26]. Likewise, an observational study conducted in Debre Berhan Referral Hospital reported an overall compliance of only 22%, suggesting a persistent gap in IPC practices among healthcare workers [27]. While only 23.9% of HCWs reported consistently wearing a face mask, this has important implications. An Ethiopian study by Deressa et al. reported widespread shortages of PPE and low satisfaction with PPE availability among HCWs, suggesting that institutional resource constraints may limit consistent mask use [28]. Likewise, Tekalegn et al. found that HCWs who had received PPE training were more than twice as likely to correctly use facemasks, highlighting the importance of continuous training in improving compliance [29]. Possible explanations for the above finding might include inadequate implementation of IPC programs, limited availability of hand hygiene resources and personal protective equipment, insufficient refresher training, high clinical workload, and reduced risk perception of MVD.

Modifying their working habits due to fears of getting MVD was found to be associated with the practice of MVD, which revealed that HCWs who modified their working habits because of fears about getting MVD had higher odds of good practice toward MVD as compared to their counterparts. This finding suggests that perceived susceptibility to infection may motivate healthcare workers to adopt preventive behaviors and adhere more closely to recommended IPC measures [30, 31].

Modification of working habits because of concern about MVD was associated with good practice in the adjusted analysis. Healthcare workers reporting modified working habits because of concern about MVD had higher odds of good practice than those who did not report such modification. This finding should be interpreted cautiously because of the cross‐sectional design. The temporal direction of the association cannot be established, and reported modification of working habits may overlap with behaviors captured in the practice outcome. Therefore, the association may reflect a relationship between perceived occupational risk and preventive behavior rather than evidence that fear caused better practice. Previous studies similarly reported associations between perceived infection risk and infection prevention behaviors among healthcare workers [32–35], considering design and measurement differences.

An adjusted association was observed between MVD knowledge and practice. Healthcare workers with good knowledge had twice the odds of good practice compared with those with poor knowledge. This association remained statistically significant after adjusting for variables in the multivariable model. However, because knowledge and practice were measured simultaneously in this cross‐sectional study, the finding does not establish that greater knowledge caused better practice. Reverse causality is possible, whereby healthcare workers with greater engagement in infection prevention practices may have greater exposure to MVD‐related information and higher knowledge scores. Nevertheless, the finding supports strengthening knowledge as a component of preparedness interventions, while recognizing that training alone may not ensure appropriate practice in the absence of adequate resources, supervision, and institutional support.

This finding aligns closely with several multicountry and regional studies on MVD and related VHFs in sub‐Saharan Africa. A comprehensive cross‐sectional study evaluating MVD knowledge, attitudes, and practices across eight African nations found that correctly identifying transmission routes was heavily associated with positive behavioral shifts and modified habits [14]. A study of Nigerian healthcare workers found MVD knowledge and preparedness differences across professions, with doctors showing greater knowledge but lower confidence in hospital preparedness than nurses [36]. These findings suggest that knowledge and perceived preparedness may vary across professional roles, although the Nigerian study did not establish a causal or correlational relationship between knowledge and clinical preparedness. This also underscores that health literacy is fundamental to epidemic preparedness, serving as a strategic determinant that shapes risk perception, preventive behavior, and community resilience during health crises [37, 38].

### 4.1. Recommendations

Based on the observed knowledge gaps, self‐reported infection prevention practices, and practice‐associated factors, the following actions are recommended:

1. Competency‐based MVD and VHF training: Dilla University Teaching Hospital should provide competency‐based training covering suspected MVD case recognition, clinical presentation, transmission routes, zoonotic and environmental risk factors, notification procedures, and IPC.
2. Simulation‐based preparedness: Periodic simulation exercises should strengthen healthcare workers’ abilities to triage, identify, notify, isolate, and appropriately refer suspected MVD/VHF cases.
3. Availability and correct use of PPE: Hospitals should ensure continuous PPE availability for VHF response and provide training and competency assessments on donning, use, and doffing procedures.
4. Hand hygiene and IPC auditing: Observed gaps in hand hygiene practices, the facility should conduct periodic audits of hand hygiene and IPC competencies and provide feedback to healthcare workers. Access to hand hygiene supplies must be ensured.
5. Safe specimen handling and waste management: Laboratory, clinical, and support healthcare workers should receive training on safe specimen collection, packaging, transport, processing, and waste management during suspected VHF events.
6. Facility preparedness and referral pathways: Hospitals should maintain an updated MVD/VHF preparedness plan defining triage, notification, isolation, referral, communication, and coordination procedures.
7. Integrated preparedness approach: Preparedness should extend beyond hospital‐based IPC to include environmental and zoonotic risk, detection, surveillance, diagnosis, supportive treatment, and coordination with public health authorities. Where an MVD vaccine becomes available and is recommended by national/international authorities, vaccination should be incorporated into outbreak preparedness and response strategies as appropriate.
8. Further research: Multicenter studies using standardized/criterion‐referenced KAP measures and observational assessments of IPC competency and facility readiness are needed to determine whether self‐reported practices correspond to actual preparedness.

### 4.2. Strength and Limitation

This study provides facility‐level evidence on knowledge, attitudes, practices, and factors associated with MVD practice among healthcare workers in a southern Ethiopian teaching‐hospital setting. The 98.3% response rate supports completeness of collected responses, and a previously developed questionnaire provided a structured approach to KAP assessment. However, limitations remain. First, the cross‐sectional design prevents determination of temporal or causal relationships between knowledge, attitudes, perceived risk, and practice. Second, the study was conducted at a single teaching hospital, potentially limiting generalizability to other healthcare facilities and regions of Ethiopia. Third, self‐reported practice measures may have introduced recall and social‐desirability bias, potentially over‐reporting infection prevention behaviors. Fourth, the practice items assessed general infection prevention behaviors, such as hand hygiene, face mask and glove use, rather than MVD‐specific operational preparedness activities such as triage, isolation, notification, use of VHF‐appropriate PPE, specimen handling, waste management, and safe burial procedures. Therefore, the findings should not be interpreted as a comprehensive measure of MVD outbreak preparedness. Fifth, the study did not assess practical IPC competencies or the availability and accessibility of PPE and preparedness resources. Sixth, the analysis focused on factors associated with practice, not knowledge or attitude. Finally, study‐specific mean cutoffs for classifying knowledge, attitude, and practice limit comparability with studies using standardized or criterion‐referenced thresholds.

## 5. Conclusions

This study assessed knowledge, attitudes, and practices regarding MVD among healthcare workers at Dilla University Teaching Hospital, southern Ethiopia. Although over half of participants were classified as having good knowledge, positive attitudes, and good practice based on study‐specific mean cutoffs, important gaps emerged, particularly in environmental/zoonotic risk factors and selected infection prevention practices. Good knowledge and working‐habit modification due to concern about acquiring MVD were positively associated with good practice after multivariable adjustment. Given the cross‐sectional design, findings indicate associations rather than causality. Strengthening competency‐based MVD training, simulation‐based preparedness, access to PPE and hand‐hygiene resources, IPC competency auditing, safe specimen/waste management, and facility preparedness and referral systems may improve readiness for future MVD and other VHF events.

**Nomenclature**
HCW: Healthcare worker
IPC: Infection prevention control
KAP: Knowledge, attitude, and practice
MVD: Marburg virus disease
VHF: Viral hemorrhagic fevers

## Author Contributions

Abel Desalegn Demeke well designed the study, analysis, interpretation, writing, and also prepared and critically evaluated the manuscript. Abel Desalegn Demeke, Wondimagegn Wolde Eba, Alem Bayable, Alemayehu Yeshiwas, Takla Tamir, Aboma Tsegaye Molatu, Mesfin Abebe, Endris Seid Amede, Mequanint Ayehu Akele, Samuel Jigso Dube, Migbaru Endawoke, and Atirsaw Shimekaw Goshu participated in the review proposal, data analysis, interpretation, and write‐up. They also made critical revisions to the text.

## Funding

No funding was received for this manuscript.

## Disclosure

The final manuscript was read and approved by all authors.

## Ethics Statement

This study was conducted per ethical standards laid down in the 1964 Declaration of Helsinki and its later amendments or comparable ethical standards [39]. Ethical approval was obtained from the institutional review board of Dilla University with the unique registration number duirb/22/18 prior to the conduction of the survey. All respondents were provided online written informed consent before starting a survey through a question that ensured their willingness to participate (if you want to participate in the study, press agree). In addition, they were informed they have the right to withdraw from the survey at any time before its completion. Furthermore, we were assured that all data would only be used for the purpose of research, be anonymous, and be confidential.

## Consent

Please see the Ethics Statement.

## Conflicts of Interest

The authors declare no conflicts of interest.

## Supporting Information

Additional supporting information can be found online in the Supporting Information section.

## Supporting information


**Supporting Information** STROBE checklist.

## Data Availability

The data that support the findings of this study are available on request from the corresponding author. The data are not publicly available due to privacy or ethical restrictions.

## References

[1] WHO , Marburg Virus 6 Disease: Key Facts, 2021.

[2] CDC , Marburg Virus Disease, 2024, CDC.

[3] Brainard J. , Hooper L. , Pond K. , Edmunds K. , and Hunter P. R. , Risk Factors for Transmission of Ebola or Marburg Virus Disease: A Systematic Review and Meta-Analysis, International Journal of Epidemiology. (2016) 45, no. 1, 102–116, 10.1093/ije/dyv307. PMC4795563 26589246

[4] Chakraborty S. , Chandran D. , Mohapatra R. K. et al., Marburg Virus Disease-A Mini-Review, 2022.

[5] WHO , Equatorial Guinea Confirms First-Ever Marburg Virus Disease Outbreak, 2023, WHO.

[6] CDC , Marburg Virus Disease Outbreaks, 2023, CDC.

[7] Orrall A. , Large Marburg Virus Disease Outbreak in the Republic of Rwanda, JAMA. (2024) 332, no. 20, 10.1001/jama.2024.22057. 39485361

[8] Who , Disease Outbreak News, Marburg virus disease in Rwanda. (2024) .

[9] Mohapatra R. K. , Sahu A. R. , Mishra S. , Tiady S. K. , and Tuglo L. S. , Tanzania Confirms Its Second MVD Outbreak: A Constantly Emerging Outbreak Calls for Effective Countermeasures in Light of One Health Approach and Preparedness to Counter Future Epidemics, One Health. (2025) 20, 10.1016/j.onehlt.2025.101070. PMC12142521 40486756

[10] Kortepeter M. G. , Dierberg K. , Shenoy E. S. , and Cieslak T. J. , Marburg Virus Disease: A Summary for Clinicians, International Journal of Infectious Diseases. (2020) 99, 233–242, 10.1016/j.ijid.2020.07.042. PMC7397931 32758690

[11] MoHEaEPH I. , Ethiopian National Guidelines for Case Management and Facility Readiness of Marburg Virus Disease, MOH-Ethiopia and EPHI. (2025) .

[12] Raab M. , Pfadenhauer L. M. , Millimouno T. J. , Hoelscher M. , and Froeschl G. , Knowledge, Attitudes and Practices Towards Viral Haemorrhagic Fevers Amongst Healthcare Workers in Urban and Rural Public Healthcare Facilities in the N’Zérékoré Prefecture, Guinea: A Cross-Sectional Study, BMC Public Health. (2020) 20, no. 1, 10.1186/s12889-020-8433-2. PMC7059383 32138720

[13] Hersi M. , Stevens A. , Quach P. et al., Effectiveness of Personal Protective Equipment for Healthcare Workers Caring for Patients With Filovirus Disease: A Rapid Review, PLoS One. (2015) 10, 10.1371/journal.pone.0140290. PMC4599797 26451847

[14] Hussein M. F. , Gebreal A. , Saleeb M. et al., Knowledge, Attitudes and Practices of the General Population Towards Marburg Virus Disease in Sub-Saharan African Countries: A Cross-Sectional Study, Transactions of the Royal Society of Tropical Medicine and Hygiene. (2025) 119, no. 4, 354–366, 10.1093/trstmh/trae122. 39698799

[15] Kangbai J. B. , Senesis T. , Juana J. , Team E. , and Team W. , An Assessment of the Knowledge, Attitude, and Practice Towards Ebola and Marburg Fever Amongst Residents in Kailahun District, Sierra Leone, Annals of Medical and Health Sciences Research. (2022) 12.

[16] Yadav S. , Shivangi Y. , Yadav R. K. , Narang R. K. , and Singh A. , Marburg Virus Disease in Ethiopia: the First National Outbreak and Its Public Health Implication, Future Virology. (2026) 21, no. 5, 307–311, 10.1080/17460794.2026.2677357.

[17] Kebede A. G. , Aynalem Z. B. , Tadele A. E. et al., Knowledge, Attitudes, and Preparedness Regarding Marburg Virus Disease Among Healthcare Workers in Awi Zone Public Hospitals, Northwest Ethiopia: A Multicenter Cross-Sectional Study, Tropical Medicine and Infectious Disease. (2026) 11, no. 5, 10.3390/tropicalmed11050125. PMC13211424 42188854

[18] Laikemariam M. , Fentie E. , and Yenesew A. , Determinants of Healthcare Workers’ Attitudes Towards Marburg Virus Disease Outbreak Response in Northwest Ethiopia: A Cross-Sectional Study, BMC Public Health. (2026) . 10.1186/s12889-026-27885-1 PMC13378065 42177442

[19] Nyakarahuka L. , Skjerve E. , Nabadda D. et al., Knowledge and Attitude Towards Ebola and Marburg Virus Diseases in Uganda Using Quantitative and Participatory Epidemiology Techniques, PLoS Neglected Tropical Diseases. (2017) 11, no. 9, 10.1371/journal.pntd.0005907. PMC5608436 28892520

[20] Lukman L. , Tawakalitu A. J. , Patrick A. et al., Concurrent Emergence of Marburg Virus Disease in Tanzania and Equatorial Guinea: A Ticking Time Bomb, 2023, LWW.

[21] Abebe T. B. , Bhagavathula A. S. , Tefera Y. G. et al., Healthcare Professionals’ Awareness, Knowledge, Attitudes, Perceptions and Beliefs About Ebola at Gondar University Hospital, Northwest Ethiopia: A Cross-Sectional Study, Journal of Public Health in Africa. (2016) 7, no. 2, 10.4081/jphia.2016.570. PMC5345406 28299161

[22] Adewole D. A. , Bello S. , Olowookere S. A. , and Olarewaju S. , Healthcare Workers’ Awareness and Perception of Infection Prevention and Control Policies and Practices Amidst Viral Haemorrhagic Fever Epidemics in Nigeria: A Qualitative Study, Canadian Journal of Infection Control/Revue Canadienne de Prévention des Infections. (2016) 31.

[23] Ilesanmi O. S. , Afolabi A. A. , and Atoyebi A. O. , Beyond the COVID-19 Pandemic in Nigeria: Improving Awareness and Preparedness for Managing Viral Haemorrhagic Fevers, Journal of Interventional Epidemiology and Public Health. (2024) 7, 1–8.

[24] Ryamukuru D. , Mukantwari J. , Uwingabire F. , and Nyiringango G. , Marburg Virus Disease in Rwanda: The Role of Infection Prevention and Control in Reducing Transmission of Infectious Disease Outbreaks Among Healthcare Professionals, Rwanda Journal of Medicine and Health Sciences. (2025) 8, no. 1, 139–143, 10.4314/rjmhs.v8i1.11. PMC12188265 40575275

[25] Mehta V. , Negi S. , Mathur A. et al., Knowledge, Attitude, and Perception About Marburg Virus in Healthcare Workers of India, Discover Public Health. (2024) 21, no. 1, 10.1186/s12982-024-00155-4.

[26] Soboksa N. E. , Negassa B. , Kanno G. , Ashuro Z. , and Gudeta D. , Hand Hygiene Compliance and Associated Factors Among Healthcare Workers in Ethiopia: A Systematic Review and Meta‐Analysis, Advances in Preventive Medicine. (2021) 2021, 7235248–10, 10.1155/2021/7235248. PMC8692043 34950518

[27] Kolola T. and Gezahegn T. , A Twenty-Four-Hour Observational Study of Hand Hygiene Compliance Among Health-Care Workers in Debre Berhan Referral Hospital, Ethiopia, Antimicrobial Resistance and Infection Control. (2017) 6, no. 1, 10.1186/s13756-017-0268-y. PMC5663127 29093813

[28] Deressa W. , Worku A. , Abebe W. , Gizaw M. , and Amogne W. , Availability and Use of Personal Protective Equipment and Satisfaction of Healthcare Professionals During COVID-19 Pandemic in Addis Ababa, Ethiopia, Archives of Public Health. (2021) 79, no. 1, 10.1186/s13690-021-00668-3. PMC8369137 34404464

[29] Tekalegn Y. , Sahiledengle B. , Bekele K. et al., Correct Use of Facemask Among Health Professionals in the Context of Coronavirus Disease (COVID-19), Risk Management and Healthcare Policy. (2020) 13, 3013–3019, 10.2147/rmhp.s286217. PMC7755342 33376425

[30] Mutsonziwa G. A. , Mojab M. , Katuwal M. , and Glew P. , Influences of Healthcare Workers’ Behaviours Towards Infection Prevention and Control Practices in the Clinical Setting: A Systematic Review, Nursing Open. (2024) 11, no. 3, 10.1002/nop2.2132. PMC10941556 38488425

[31] Benishek L. E. , Radonovich L. J. , Blackley B. H. , and Weissman D. N. , Healthcare Workers’ Infection Risk Perceptions of Aerosol-Generating Procedures and Affective Response, Antimicrobial Stewardship & Healthcare Epidemiology. (2023) 3, no. 1, 10.1017/ash.2022.276. PMC9972538 36865705

[32] Selvaraj S. A. , Lee K. E. , Harrell M. , Ivanov I. , and Allegranzi B. , Infection Rates and Risk Factors for Infection Among Health Workers During Ebola and Marburg Virus Outbreaks: A Systematic Review, Journal of Infectious Diseases. (2018) 218, no. suppl_5, S679–S689, 10.1093/infdis/jiy435. PMC6249600 30202878

[33] Jones C. L. , Jensen J. D. , Scherr C. L. , Brown N. R. , Christy K. , and Weaver J. , The Health Belief Model as an Explanatory Framework in Communication Research: Exploring Parallel, Serial, and Moderated Mediation, Health Communication. (2015) 30, no. 6, 566–576, 10.1080/10410236.2013.873363. PMC4530978 25010519

[34] Raven J. , Wurie H. , and Witter S. , Health Workers’ Experiences of Coping With the Ebola Epidemic in Sierra Leone’s Health System: A Qualitative Study, BMC Health Services Research. (2018) 18, no. 1, 10.1186/s12913-018-3072-3. PMC5887191 29622025

[35] Nevez G. , Le Gal S. , Noel N. et al., Investigation of Nosocomial Pneumocystis Infections: Usefulness of Longitudinal Screening of Epidemic and Post-Epidemic Pneumocystis Genotypes, Journal of Hospital Infection. (2018) 99, no. 3, 332–345, 10.1016/j.jhin.2017.09.015. 28943270

[36] Oisakede E. O. , Asogun D. , Otaigbe O. et al., Assessment of Healthcare Worker Preparedness and Health Literacy for Marburg Virus Disease in Nigeria: A Cross-Sectional Study, Southern African Journal of Infectious Diseases. (2026) 41, no. 1, 10.4102/sajid.v41i1.764. PMC12869813 41647323

[37] Lamsir S. , Community Preparedness for Health Disasters: A Study of Community Health Literacy, Oshada. (2025) 2, no. 6, 165–176, 10.62872/4dj2mv87.

[38] Suwarningsih S. , Community Preparedness for Health Disasters: A Community Health Literacy Study, Oshada. (2025) 2, no. 6, 220–230, 10.62872/wdjt9b66.

[39] The declaration of Helsinki , The Oxford Textbook of Clinical Research Ethics, 2008, Ashcroft RE.

